# Bio-Functional Properties of Lactic Acid Bacteria in Functional Foods

**DOI:** 10.3390/foods15030528

**Published:** 2026-02-03

**Authors:** Svetoslav Dimitrov Todorov

**Affiliations:** 1 ProBacLab, Laboratório de Microbiologia de Alimentos, Departamento de Alimentos e Nutrição Experimental, Food Research Center, Faculdade de Ciências Farmacêuticas, Universidade de São Paulo, São Paulo 05508-000, SP, Brazil; slavi310570@abv.bg; Tel.: +55-11-9-63062012; 2 CISAS—Center for Research and Development in Agrifood Systems and Sustainability, Instituto Politécnico de Viana do Castelo, 4900-347 Viana do Castelo, Portugal; 3 Department of General Hygiene, Sechenov First Moscow State Medical University, Trubetskaya Str. 8, Bldg. 2, Moscow 119991, Russia

Lactic acid bacteria (LAB) are pivotal in the production of fermented foods, serving as functional ingredients that enhance both the quality and safety of these products. Fermentation, a process revered for its preservation capabilities, also improves the sensory, technological, and nutritional attributes of raw ingredients. LAB are instrumental in this transformation, contributing to the development of desired sensory properties and ensuring microbiological safety. Moreover, in traditional fermented foods, LAB initiate fermentation by converting carbohydrates into lactic acid, which acts as a natural preservative. This process not only extends the shelf life of food products but also enriches them with beneficial bacteria that promote gut health. The presence of LAB in fermented foods like cheese, yogurt, sauerkraut, salami, olives, kimchi, etc., has been associated with improved digestibility, enhanced nutrient bioavailability, and potential therapeutic benefits. Selected LAB can play a crucial role in the cultural identity of regional cuisines, often involving native ingredients and traditional practices. The variability of artisanal production, however, poses challenges in identifying specific LAB strains, which can include species like lactobacilli and streptococci, among others, that can be actively involved not only technologically and in terms of bio-preservation, but also have an effect on their therapeutic properties. Despite this, the health benefits conferred by these microorganisms, such as improved lactose digestion, infection prevention, and possible cancer risk reduction, underscore their significance in the food industry.

LAB are an essential part of everyday life. They serve as starter cultures; are involved in the technological transformations of raw material into fermented food products; have biotechnical applications as producers of enzymes, polysaccharides, antimicrobials, or other bioactive metabolites with industrial and/or health importance; have been explored as potential probiotics or essential factories, transforming prebiotics into functional metabolites; and, even when dead, serve as postbiotics with an essential role in the improvement of human and animal health. LAB are essential functional ingredients in fermented foods, offering a multitude of benefits, from enhancing flavor profiles to promoting health and well-being. Their role in food fermentation underscores the fascinating intersection between culinary tradition and modern nutritional science.

This Special Issue presents a collection of diverse manuscripts with a common underlying focus: the bio-functional properties of LAB in functional foods. Having received numerous academic and technical contributions from authors, the editorial team at *Foods* carried out an intensive evaluation process and applied a thorough peer review procedure, leading to the final selection of 11 outstanding papers to comprise the current edition, which aims to present a collection of work reflecting the beneficial properties of the diverse universe of LAB. The featured contributors include scientists from Brazil, Portugal, the Republic of Korea, China, and Italy. The first featured contribution focuses on the beneficial properties of LAB, emphasizing the applications of starter cultures. In the second paper, the impact of sucrose as a glucosyl donor and both maltose and *Artemisia capillaris* as acceptors on gut microbiota is investigated through fecal fermentation, and the role of the *Leuconostoc mesenteroides* strain MKSR in these processes is further analyzed. Following this, the third contribution addresses new trends in bioinformatics and bacteriocin mining in the case of using *Lactiplantibacillus pentosus* strain PCZ4 to screen for new broad-spectrum antimicrobial peptides with potential biopreservative properties in snakehead fish. The fourth contribution analyses the beneficial effect of *Lacticaseibacillus paracasei* L9 on oral microbiota and for the potential control of cariogenic factors in *Streptococcus mutans*, with validation using a mouse model. Investigating in vitro, ex vivo, and in vivo evidence of nitrate-reducing activity in *Levilactobacillus brevis* strain CD2, the fifth contribution considers the potential of this strain as an effective tool in oral and systemic health applications. The sixth contribution presents an overview of the crossing probiotic and antimicrobial properties of LAB isolated from different plants. Following this, the seventh contribution investigates the beneficial effect of purple-fleshed sweet potato lyophilized powder on the physicochemical and textural properties, LAB viability, and microstructure of stirred yogurt, where traditional knowledge and modern methods intersect, improving our knowledge on the interactions between prebiotics and beneficial LAB cultures. Selecting LAB with antifungal properties, the eighth featured paper explores their impact on the quality and shelf life of rye bran sourdough bread. Investigating the health-promoting properties of *Bifidobacterium animalis*, the ninth paper in the collection details findings that supplementation can improve intestinal barrier function and alleviate antibiotic-associated diarrhea, demonstrated using a mouse model. Despite not being LAB, representatives from this genus have long been shown to have beneficial effects on both human and animal health, prompting this investigation. In the tenth featured paper, evidence is presented suggesting that the supplementation of camel milk with a probiotic can improve the levels of serum glucose and cholesterol, as well as the related cytokines, in patients with type 2 diabetes mellitus. Here, the natural benefits of the dairy product and the beneficial cultures are shown to synergistically interact to deliver health-promoting benefits. In the final study included in this collection, the authors provided evidence that the AraR transcription factor affects the sugar metabolism and acid tolerance of *Lactiplantibacillus plantarum*. This marks a new discovery regarding the metabolic specificity and intimate processes of microbial behavior, as well as the beneficial effects that may arise as a result. Despite the range of perspectives presented in this Special Issue, throughout it, the beneficial properties of LAB remain the central focus, along with the exploration of their bioactive metabolites and functional properties.

LAB have a reputation as beneficial microbes, with several of them having been characterized and explored by the food industry [1] and applied as powerful probiotics [2] and postbiotics [3] cultures. Numerous LAB have also been granted GRAS status and are considered as the natural microbiota of various fermented (traditional and industrial) food products [4], as well as part of natural human and animal microbiota [1]. However, other (and no small amount) LAB are spoilage [5] opportunists and effective pathogens [6]. A prime example is genus *Streptococcus*, of which only three species—*St. thermophilus*, *St. salivarius*, and *St. macedonicus*—are considered safe, according to the bulletin of the International Dairy Association [7]. The remaining representatives of *Streptococcus* are pathogenic for humans and animals [8]. Another example of a non-safe LAB is *Enterococcus* spp., where vancomycin-resistant strains pose a serious threat in health care facilities [9]. Moreover, despite having traditionally been considered safe, *Lactiplantibacillus plantarum* strains have been described as health-affecting cultures [10]. The safety of LAB is an essential point that merits attention in all investigations exploring the beneficial properties of new microbial cultures. Investigations of this issue must form part of the research protocols and cannot be neglected. It is important to note that since 2020, marking a change in the taxonomy of lactobacilli, the taxonomic status of many LAB has been updated [11], with appropriate abbreviations for their presentation in the scientific literature being proposed in 2023 [12]. However, this discussion remains largely outside the remit of this Special Issue, which aimed to explore the functional properties of LAB.

In their work, Carneiro et al. [Contribution 1] present an overview of LAB with industrial importance related to meat products. Focusing on the main characteristics of LAB, they afford special emphasis to their nutritional, functional, and technological benefits. In addition to this, particular attention was paid to the importance of beneficial metabolites that can be linked to the metabolic specificity of LAB associated with the fermentation process. Those listed in this review paper [Contribution 1] include peptides with antimicrobial, antidiabetic, antihypertensive, and immunomodulatory properties. While their health-promoting benefits were discussed, this was kept within the context of their essential importance from a technological point of view. The authors highlight antimicrobial peptides, GABA, exopolysaccharides, antioxidants, and vitamins as beneficial metabolites that influence safety, technological processes, and even provide health-promoting consumer benefits. Additional emphasis was given to the potential hazards related to some LAB that may present virulence properties, requiring a critical evaluation of the use of specific strains in food formulations.

The second contribution, that of Moon et al., reports on the impact of a transglycosylated product (ACOD) catalyzed by *Leuconostoc mesenteroides* MKSR dextransucrase using sucrose as a glucosyl donor and both maltose and *Artemisia capillaris* as acceptors on gut microbiota through fecal fermentation. This foundational research highlights the ability of ACOD to promote the growth of probiotics strains belonging to beneficial lactobacilli, including representatives such as strains from species *Lactiplantibacillus plantarum*, *Lacticaseibacillus casei*, *Lacticaseibacillus rhamnosus* GG, and *Leuconostoc mesenteroides* MKSR, whilst simultaneously presenting inhibitory properties associated with the growth of pathogenic bacteria, including *Escherichia coli* (particularly *E. coli* O157:H7), *Enterococcus faecalis*, *Listeria monocytogenes*, *Staphylococcus aureus*, *Shigella flexneri*, *Streptococcus mutans*, *Pseudomonas aeruginosa*, and *Bacillus cereus* during independent cultivation. The authors provide evidence that in a 24 h fermentation process, ACOD can greatly increase the production of short-chain fatty acids (SCFAs) compared to the negative control experiment, where fructooligosaccharide (FOS) was applied as a control group. It is reported that ACOD led to an increase as great as 4.5-fold in acetic acid production compared to the effect of FOSs, which was investigated in a parallel experimental set-up, and a 3.3-fold increase in propionic acid production. However, it was observed that the inclusion of both ACOD and FOS resulted in higher levels of butyric acid than the blank. In particular, ACOD notably modulated the composition of the gut microbiota, where results pointed to an increase in the relative abundances of lactobacilli and, more importantly, a decrease in *Escherichia*/*Shigella* and *Salmonella*. However, although demonstrated to be an excellent prebiotic, FOS cannot be considered a universally beneficial carbohydrate. It was observed that FOS can promote the growth of *Salmonella*. The authors therefore suggest that ACOD can be considered as a candidate for application as a prebiotic, improving the intestinal environment by being specifically and actively used by beneficial bacteria.

Du et al. [Contribution 3] reported on the production of antimicrobial peptides by *Lactiplantibacillus pentosus*, a strain designated as PCZ4, isolated from kimchi, which displayed interesting broad-spectrum antibacterial activity, with potential biopreservative effects on snakehead fish. Interestingly, in the work of Du et al. [Contribution 3], bioinformatic approaches were applied to screen for potential producers of antimicrobial peptides. Taking in consideration that *Lactiplantibacillus pentosus* PCZ4 presenting a broad spectrum of antimicrobial activity, the authors subjected it to whole-genome sequencing, including screening of its chromosome and three plasmids. Based on bioinformatic analysis through BAGEL4 mining, the authors successfully mapped the presence of classes IIa and IIb bacteriocins, as well as the genetic determinants encoding the potential production of plantaricin S. Moreover, two new antibacterial peptides, Bac1109 and Bac2485, were predicted from scratch by limiting open reading frames. Furthermore, in this work [Contribution 3], the authors observed that the application of PCZ4 crude extract during the refrigerated storage of snakehead fish improved the microbiological status of the product, contributed to a reduction in the total bacterial count, slowed the increase in TVB-N and pH values, improved the sensory quality of the snakehead, and extended its shelf life by 2 days. As a result, the authors concluded that crude extract from *Lpb. pentosus* PCZ4 can effectively inhibit the growth of *Aeromonas hydrophila* in an artificially contaminated snakehead fish.

The application of antimicrobial peptides has not been exclusively considered for food biopreservation applications. In recent decades, the beneficial role of bacteriocins has been actively explored in health-promoting sectors [13], with studies pointing to their essential role in combating clinically important pathogens [14] and even providing evidence for synergistic interactions between bacteriocins and antibiotics [15] or the *quorum sensing* role of bacteriocins in the control of human and animal pathogens [16]. Pu et al. [Contribution 4] reported the results of an animal model study, where the role of *Lacticaseibacillus paracasei* L9 as a potential candidate for control of the oral cavity microbiota and cariogenic factors in *Streptococcus mutans* was studied. It is a well-known fact that *S. mutans* plays a central role in oral hygiene, where it is associated with dental integrity. Its biofilm formation properties, related to its extracellular polysaccharides, can strongly facilitate the persistence of bacteria on the tooth surface by creating a stable living environment and hindering their removal by natural defense substances in the oral cavity, such as saliva. However, in the oral microbiota, additional beneficial strains and dietary habits have the potential to influence the reduction or removal of *S. mutans*. In this study [Contribution 4], the authors employed a BALB/c mouse model to explore the protective effects of *Lbs. paracasei* L9 on dental caries. Mice were introduced to *S. mutans* and subsequently treated with *Lbs. paracasei* L9 or *S. salivarius* K12 for 28 consecutive days. It was reported that *Lbs. paracasei* L9 significantly ameliorated early enamel caries, and both *Lbs. paracasei* L9 and *S. salivarius* K12 cooperatively downregulated the expressions of critical cariogenic factors, effectively suppressing the initial adhesion of *S. mutans* and the formation of dental plaques [Contribution 4]. Moreover, it was shown that *Lbs. paracasei* L9 was able to reshape the oral microbiota of caries-affected mice, selectively reducing the abundance of pathogens and augmenting the abundance of probiotics such as Lactobacillaceae and *S. salivarius*. It should be stated that, as this study [Contribution 4] was conducted at the pilot stage, and all results were observed in an animal model, appropriate clinical studies should be planned and performed in order to confirm the beneficial role of *Lbs. paracasei* L9 as an effective anti-caries agent in humans; however, the current study offers a strategic innovative approach for the management of dental caries, clearly highlighting the potential of beneficial strains/probiotics in the field of oral health.

In their contribution, Altamura et al. [Contribution 5] evaluated oral health issues from different aspects, where in vitro, ex vivo, and in vivo evidence of nitrate-reducing activity in *Levilactobacillus brevis* CD2 was reported for their application as a potential tool for oral and systemic health. The authors [Contribution 5] provided evidence in support of their hypothesis that the use of nitrate-reducing bacterial strains as probiotics can contribute to enhancing the benefits of nitrate metabolism for both oral and systemic health. In this study [Contribution 5], the authors explored the nitrate reductase activity of *Lvb. brevis* CD2 (DSM-27961/CNCM I-5566), a strain that has been widely applied as a starter culture in fermented foods and recognized for its multifaceted health-promoting probiotic properties. Exploring whether the probiotic lysate could enhance nitrate reduction in conditions ex vivo, the study aimed to further investigate the connection between lactate metabolism and nitrite production. The authors noted that salivary D-lactate levels after 3 h, with or without *Lvb. brevis*, may indicate a beneficial effect of the probiotic. It was shown that *Lvb. brevis* CD2 exhibits significant intrinsic and concentration-dependent nitrate reductase activity. Moreover, they reported that treatment with *Lvb. brevis* for 3 h significantly increased nitrite generation across all saliva samples, with further enhancement observed after the addition of exogenous nitrates. It was reported that *Lvb. brevis* CD2 also significantly improved salivary pH and buffering capacity, particularly when combined with nitrate. Consequently, it was concluded that treatment with probiotic *Lvb. brevis* CD2 can reduce D-lactate in saliva. Moreover, the authors [Contribution 5] validated their observations in in vitro and ex vivo positive results by exploring oral nitrate-reducing activity in saliva samples from healthy individuals treated for four weeks with *Lvb. brevis* CD2 lozenges. It is important to emphasize that the results indicated that the probiotic-treated group showed a significant increase in oral nitrate-reducing capacity compared to baseline and placebo groups after four weeks of treatment. In conclusion, it was suggested that *Lvb. brevis* CD2 acts as a nitrate-reducing probiotic, providing new insights into its health benefits and complementing findings from previous studies.

Sharma and Lee [Contribution 6] reported on probiotics with antimicrobial properties isolated from different plant materials. Historically, it was recommended that probiotics be obtained from the same eukaryotic species as where they will be applied [17]. For example, probiotics for human applications should be isolated from healthy humans. This concept is correct to some extent, since the probiotic bacterial cultures will have a better survival rate as they are already adapted to the environment in which they will be applied. However, in the last few decades, numerous reports have shown that probiotics can be selected from different ecological environments, including from inhabitants of extreme conditions, and can be appropriately applied as probiotics for humans and other animals [17]. LAB are a heterogeneous group of bacteria, inhabiting different parts of humans, animals, plants, and soil, and are present in fermented food products, with several of them being described as health-promoting microorganisms [Contribution 6]. In their review, the authors [Contribution 6] investigated LAB collected from various unconventional sources—including fruits, seeds, fruit pulp, leaves, roots, vegetables, grasses, and flowers—by examining their antibacterial, antifungal, and antiviral characteristics. Special attention was given to different LAB, including representatives from genera such as *Lactobacillus* (according to the meaning of the taxonomic norms before the changes in 2020 by Zhang et al. [11]), *Leuconostoc*, *Weissella*, *Enterococcus*, *Pediococcus*, *Bacillus*, and *Fructobacillus*. The authors [Contribution 6] examined the properties of these cultures as probiotics, postbiotics, and paraprobiotics, and highlighted antimicrobial mechanisms such as the secretion of bacteriocins, reuterin, organic acids, peptides, exopolysaccharides, and hydrogen peroxide. Focusing on the application of LAB, they highlighted their production of antimicrobial metabolites that contribute to food preservation, safety, and medicinal uses, while emphasizing the need for evidence confirming their safety.

In their study, Cunha Junior et al. [Contribution 7] evaluated the beneficial properties of purple-fleshed sweet potato lyophilized powder and its effect on the modulation of the physicochemical properties, LAB viability, microstructure, and textural attributes of stirred yogurt. Sweet potatoes are known for their nutritional and prebiotic characteristics [3]. The authors aimed to investigate whether lyophilized powder made from purple-fleshed sweet potatoes could serve as a versatile ingredient to enhance both the composition and quality of stirred yogurts. They reported the physical and chemical properties, color, monomeric anthocyanin content, LAB viability, water retention capacity, microstructure, and texture in an experimental strategy where basic yogurts was enriched with the lyophilized powder of purple-fleshed sweet potato at different levels between 2% (YLP2), 4% (YLP4), and 6% (YPL6) over a period of 30 days under refrigeration (4 °C). Researchers found that lyophilized purple-fleshed sweet potato powder can turn the yoghurt various shades of pink, which may appeal to consumers. In addition to significantly increasing water retention (*p* < 0.05) and lowering water activity—both factors important for product safety—no post-acidification occurred during storage. All explored formulations (YLP2, YLP4, and YLP6) presented relevant stability regarding the number of viable LAB cells compared to the control sample (without enrichment) during storage, an important point given the fact that fermented dairy products are expected to contain viable bacterial cells for the benefit of consumers. The addition of lyophilized purple-fleshed sweet potato powder helped enhance the microstructure of the final products, resulting in more uniform and densely cross-linked networks with fewer empty spaces, no matter the amount used [Contribution 7]. Moreover, the yogurts (YLP4 and YLP6) were firmer and creamier compared to the control. Given these findings, the authors suggest that lyophilized purple-fleshed sweet potato powder is a promising multifunctional ingredient for yogurts, serving as a natural colorant, stabilizer, emulsifier, and thickener to enhance its technological and functional properties.

The antifungal properties of LAB have recently gained scientific attention, having been only partially explored in the past. Previous authors had mapped the different metabolites produced by LAB and suggested the application of bacterial cultures or the produced metabolites for the control of fungal spoilage organisms in the food industry or against relevant human and animal pathogens [18]. Thus, the contribution of Mou et al.’s [Contribution 8] report on the screening of LAB with antifungal properties and the evaluation of their impact on the quality and shelf life of rye bran sourdough bread is of great significance. In their study [Contribution 8], *Lactiplantibacillus plantarum* G8 was pre-selected as a potential candidate due to its higher antifungal properties. As a control, a second strain of *Lpb. plantarum*, denominated as G12, displaying weaker antifungal activity, was investigated. Both strains (G8 and G12) were isolated from naturally fermented wheat sourdough. Furthermore, their impacts on bread quality and shelf life were subsequently investigated. According to Mou et al. [Contribution 8], both strains exhibited robust growth in rye bran sourdough, an important characteristic related to the fact that biopreservative culture needs to be adapted to its environmental conditions. The physicochemical properties of the final products were evaluated and compared to the blank control rye bran–wheat flour dough (RB dough). It is important to underline the observation made by the authors that the application of *Lpb. plantarum* G8 resulted in a bread presenting superior antifungal efficacy, extending its shelf life by 8 days (mold appearance at room temperature: 12 days for *Lpb. plantarum* G8 versus 4 days for RB). Mou et al. [Contribution 8] suggested that most probably, these antifungal results are related to the diverse antifungal metabolites produced by the applied starter culture. Furthermore, bread prepared with *Lpb. plantarum* G8 exhibited a significantly increased diversity and content of volatile compounds and received higher preference scores from the sensory panel [Contribution 8].

Strictly speaking, according to microbial taxonomy, bifidobacteria are not considered part of the LAB group. However, bifidobacteria are one of the most explored bacterial groups, together with LAB, with regard to their probiotic properties [11]. Thus, the contribution of Du et al. [Contribution 9], who reported on the impact of supplementation with *Bifidobacterium animalis* on the improvement of intestinal barrier function and its role in the reduction of antibiotic-associated diarrhea, shown in a mouse animal model, is of notable relevance. In the last few years, the role of probiotics in the control of antibiotic-associated diarrhea (AAD) has been suggested and evaluated [19]. However, the precise mechanisms of this effect cannot be unified with regard to all suggested probiotics with such beneficial properties. Du et al. [Contribution 9] used a mouse model of AAD induced by ceftriaxone to investigate the effects and mechanisms of *B. animalis* A6 as a potential candidate for clinical studies. The observations made by the authors suggested that *B. animalis* A6 supplementation effectively attenuated ceftriaxone-associated diarrhea in mice. Moreover, the authors observed that morphological damage to the villi and crypts following antibiotic intervention was partially restored and more neatly reorganized following the *B. animalis* A6 application. Moreover, intestinal morphology annotations uncovered a significant increase in the thickness of the mucus layer in the *B. animalis* A6-treated group compared to control animals. *B. animalis* A6 intervention upregulated mucin1, strengthened the mucus layer, and increased AQP4 and SLC26A3 expression to help restore water absorption in AAD mice. It also reduced ceftriaxone-induced damage to intestinal microbiota, supporting the survival and growth of beneficial bacteria like *Bacteroidales*, *Akkermansia*, *Bifidobacterium*, and lactobacilli.

The introduction of different dairy products is always considered a challenge in terms of consumer acceptance. However, despite being considered an “exotic” product from the perspective of European consumers, the consumption of camel milk has a long history and is a traditional product in many Asian, Middle Eastern, and North African countries [20]. The beneficial and nutritional properties of camel milk are well documented [20]. In their work, Liu et al. [Contribution 10] have investigated the supplementation of camel milk powder with a probiotic culture and further evaluated how this can improve the levels of glucose in serum and cholesterol, as well as the related cytokines, in patients with type 2 diabetes mellitus. They investigated the close association between gut microbiota and diabetes, where probiotic dairy products have been shown to play an essential have garnered scientific attention in the development of functional foods with anti-diabetic activity. Liu et al. [Contribution 10] clinically evaluated a cohort of 28 volunteers with type 2 diabetes. The volunteers received 10 g of camel milk powder supplemented with *B. animalis* A6 (BBA6) twice a day, while the placebo group received only camel milk powder. The experiment was conducted over a period of 4 weeks, and the evaluation of key clinical parameters after the intervention showed a significant decrease in fasting blood glucose, serum content of total cholesterol, and pro-inflammatory cytokines (IL-6, MCP-1). Moreover, in the probiotic camel milk powder—the *B. animalis* A6 group—the levels of irisin and osteocrin increased significantly, with the level of osteonectin increasing at the same time but without reaching a significant amount. In the probiotic camel milk powder (*B. animalis* A6) group, levels of adiponectin, resistin, lipocalin-2, and adipsin significantly decreased. Moreover, the authors [Contribution 10] analyzed the gut microbiota and observed a significant enrichment in the relative abundance of *Bifidobacterium* when compared with placebo patients, an expected observation showing the appropriate survival, multiplication, and adaptation of the studied *B. animalis* A6 strain to the human gut environment. Furthermore, higher levels in fecal concentrations of glucose-1-phosphate, conduritol b epoxide, D-arabitol, dehydroascorbic acid, and dl-p-hydroxyphenyllactic acid, accompanied by a decrease in glycine, N-acetylisatin, hydroxylamine, caprylic acid, maltotriose, and guaiacol, were found in patients receiving probiotic camel milk powder—*B. animalis* A6 group. Compared with camel milk alone, the addition of *B. animalis* A6 resulted in a significant decrease in the levels of fasting blood glucose in type 2 diabetic patients; consequently, this also had a beneficial effect on improving dyslipidemia, chronic inflammation, and skeletal muscle functions, indicating the possibility of probiotic camel milk powder as a dietary treatment that targets metabolic syndromes such as diabetes, as suggested by Liu et al. [Contribution 10].

In their contribution, Zhao et al. reported on the AraR transcription factor and its effect on the sugar metabolism and acid tolerance of *Lactiplantibacillus plantarum*. This pilot study was conducted on *Lpb. plantarum*, but such fundamental research can be scaled to other probiotics from the lactobacilli group. During the evolutionary process, the microorganism developed specific strategies to adapt to acidic environments, where sophisticated transcription factors play an essential role within their hierarchical regulatory networks. Zhao et al. [Contribution 11] performed functional characterization of the AraR transcription factor LP_RS14895 by implementing multiomics investigation approaches. The authors performed RNA sequencing and identified 40 acid-responsive targets, mainly in pentose/glucuronate interconversion and amino sugar and nucleotide sugar metabolism pathways. Moreover, performing genome-wide binding analysis via DAP-seq, they identified 1279 interaction sites, and the most significantly enriched motif was suggested to be “ARCCMATMAHC”. According to Zhao et al. [Contribution 11], AraR plays an essential role in the regulation of acid tolerance and processes for the metabolization of arabinose, glucose, fructose, ribose, mannose, and trehalose. This contribution offers key insights into microbial stress responses and presents an effective method for overcoming carbohydrate inhibition in high-acid environments.

LAB are essential in the preparation of various foods, playing an important role in fermentation processes, while metabolites produced by LAB have applications in biotechnological processes, pharmaceutical production, and applied sciences. Moreover, LAB are important players in the microbiota of humans, animals, and plants, involved in the modulation of vital processes. They can be used as probiotics and, even when dead, can contribute to health and functional processes as postbiotics. Microbial metabolites produced by LAB represent an underestimated scientific treasure whose many benefits are still to be discovered and scientifically mapped. Paracelsus postulated that any drug, behind its benefits, has the potential to act as a poison, and that all is dependent on the dose of application. The same theory can be applied to LAB and their metabolites, as their effect can be hugely dependent on the type of applied microbial culture, the dose used, and the circumstances of application. Humans (and other living creatures) are the sophisticated, complex result of a long evolutionary process, where synchronization between macro- and microorganisms has resulted in well-established synergistic interactions. It is now our responsibility to gain a better understanding of these synergistic multifunctional relations and apply microbial metabolites to our benefit, as effective tools in fermentation processes and as preventive and effective elements in medical practice.

## Data Availability

The current editorial contribution was based on the papers published in the Special Issue Bio-Functional Properties of Lactic Acid Bacteria in Functional Foods.
